# A computational model of mutual antagonism in the mechano-signaling network of RhoA and nitric oxide

**DOI:** 10.1186/s12860-021-00383-5

**Published:** 2021-10-12

**Authors:** Akila Surendran, C. Forbes Dewey, Boon Chuan Low, Lisa Tucker-Kellogg

**Affiliations:** 1 Singapore-MIT Alliance, Computational Systems Biology Programme, National University of Singapore, Singapore, Singapore; 2 Centre for Assistive Technology & Innovation, National Institute of Speech & Hearing, Trivandrum, Kerala India; 3 Biological Engineering and Mechanical Engineering Departments, Massachusetts Institute of Technology, Cambridge, MA USA; 4 Department of Biological Sciences, National University of Singapore, Singapore, Singapore; 5 Mechanobiology Institute, National University of Singapore, Singapore, Singapore; 6 University Scholars Programme, National University of Singapore, Singapore, Singapore; 7 Cancer and Stem Cell Biology, and Centre for Computational Biology, Duke-NUS Medical School, Singapore, Singapore

**Keywords:** Cytoskeleton, Nitric oxide, Dynamical systems, Bistable network, Mutual antagonism, Ultrasensitivity

## Abstract

**Background:**

RhoA is a master regulator of cytoskeletal contractility, while nitric oxide (NO) is a master regulator of relaxation, e.g., vasodilation. There are multiple forms of cross-talk between the RhoA/ROCK pathway and the eNOS/NO/cGMP pathway, but previous work has not studied their interplay at a systems level. Literature review suggests that the majority of their cross-talk interactions are antagonistic, which motivates us to ask whether the RhoA and NO pathways exhibit mutual antagonism in vitro, and if so, to seek the theoretical implications of their mutual antagonism.

**Results:**

Experiments found mutual antagonism between RhoA and NO in epithelial cells. Since mutual antagonism is a common motif for bistability, we sought to explore through theoretical simulations whether the RhoA-NO network is capable of bistability. Qualitative modeling showed that there are parameters that can cause bistable switching in the RhoA-NO network, and that the robustness of the bistability would be increased by positive feedback between RhoA and mechanical tension.

**Conclusions:**

We conclude that the RhoA-NO bistability is robust enough in silico to warrant the investment of further experimental testing. Tension-dependent bistability has the potential to create sharp concentration gradients, which could contribute to the localization and self-organization of signaling domains during cytoskeletal remodeling and cell migration.

**Supplementary Information:**

The online version contains supplementary material available at 10.1186/s12860-021-00383-5.

## Background

Mutual antagonism between two signaling pathways is a motif of higher-order regulation for building circuits [1–5]. When one competing pathway is active, it turns off the other pathway. By turning off the antagonism from the other pathway, it reinforces and stabilizes its own activity. This double-negative loop is a form of positive feedback that can allow a binary decision to be sharpened and stabilized. Sharpening information signals is crucial because signaling information would otherwise get eroded by environmental noise and by transmission through many sequential steps. Dynamical systems with mutual antagonism have been studied in several contexts, often in conjunction with bistability or oscillation.

Mutual antagonism, when combined with ultrasensitivity, is a common motif for inducing bistability [6]. Bistability literally means that a system has two stable steady-states. The stability of a steady-state means that the system re-converges to the same steady-state after minor perturbations [7]. Meanwhile, the existence of a second stable steady-state means the system can reverse its decision and converge to the opposite state, in response to significant changes from the input stimuli. Bistability has been modeled in many signaling pathways including caspase-dependent apoptosis [8], MAPK signaling [9, 10], direction sensing [11] and establishment of cell polarity [12].

There is growing evidence that bistability in the Rho GTPases network can lead to intracellular spatiotemporal patterns, governing important cellular functions like motility. Mathematical modeling suggests that MLC phosphorylation depends upon bistability between Rho kinase and myosin phosphatase [13]. Bistability in a RhoA-Rac1-PAK network was shown to drive actin cytoskeletal dynamics and cell migration switches in a study combining both modeling and experimental approaches [14]. Bistability in the GTPase network was used to qualitatively model the symmetry breaking and polarization behavior of HeLa cells as observed in microfluidic experiments [15]. In another study, spatially modulated bistability was demonstrated as Rho and Cdc42 zones formed and segregated during *Xenopus* oocyte wound healing [16]. These studies show that Rho bistability can transform initial signals and graded inputs into spatially precise and temporally co-ordinated events in cytoskeletal regulation. Therefore, we seek to explore the influences and implications of Rho dynamics in other signaling networks, such as the nitric oxide system.

RhoA and nitric oxide (NO) regulate cytoskeletal tension in opposite ways. RhoA signaling activates myosin motors [17] and is a master regulator of cellular contractility. In contrast, NO is a master regulator of relaxation and the 1998 Nobel Prize was awarded for major findings in the role of NO as a signaling molecule in vasorelaxation [18]. Many individual mechanisms of crosstalk have been documented between RhoA and NO. In vascular cell types, many mechanisms exist for the RhoA pathway and its signaling effector ROCK (Rho kinase) to antagonize eNOS and NO. eNOS is a constitutively expressed synthase of NO. Nitric Oxide Synthases (NOS) are categorized as inducible (iNOS) or constitutive (cNOS), including endothelial (eNOS) and neuronal (nNOS) [19]. ROCK decreases the stability of eNOS mRNA and thus inhibits eNOS expression [20, 21]. At the post-translational level, eNOS activity is regulated by Akt-induced phosphorylation at Serine-1177, and Akt activity is regulated by ROCK, which inhibits phosphorylation of Akt [22]. The inhibitory effects of Rho/ROCK on eNOS are well-established, as confirmed with diabetic rats in vivo [23]. Another more rapid mechanism for Rho to antagonize NO production has also been documented in experiments showing that Rho inhibition augmented shear-induced production of NO [24] . The opposite direction of effect, from NO toward RhoA, has been best studied with eNOS-NO signaling to cGMP-PKG in the vascular system [25, 26]. NO activates PKG, which is a serine-threonine kinase that phosphorylates a number of proteins regulating calcium levels and the cytoskeleton. PKG-induced phosphorylation of RhoA at Serine-188 antagonizes RhoA by blocking RhoA translocation to the membrane, which prevents its activation, and by promoting RhoA association with RhoGDI [27–29]. In mice lacking the three NOS isoforms, Rho kinase activity was elevated [30]. Most of the reported cross-talk between RhoA and NO has been antagonistic, but positive relationship has also been seen [31–34].

Prior research on the cross-talk between RhoA and NO has focused on specific elements rather than on systems level interplay. Mutual antagonism would be a logical motif for organizing RhoA and NO signaling in vivo because either extreme could be detrimental to the cell [35] or wasteful of resources [36]. However, it is unclear whether both directions of antagonism co-exist in the same system. The uncertainty arises because NO-induced relaxation has been studied primarily in vascular endothelial cell types, which have high levels of NO, while RhoA-induced contraction has been studied primarily in cell types with strong contractility such as fibroblasts. Because of this uncertainty, the first step in our study is to test experimentally for two key effects in two opposite directions, in a cell type that is neither high-NO nor highly contractile (e.g., an epithelial system). This confirms that the previous forms of antagonistic signaling co-exist, and confirms that they may be studied together in a single model instead of requiring separate endothelial and mesenchymal models. To create NO in our study, we have abstained from using NO donors, which could induce unnaturally high levels of NO, and have instead used Hepatocyte Growth Factor (HGF) to induce endogenous NO production.

After the experimental confirmation of mutual antagonism between RhoA and NO, our next goal is theoretical analysis for the functional implications of the mutual antagonism. We built a model of RhoA-NO signaling and explored whether the system is capable of bistable behavior. The model of RhoA-NO signaling includes 31 reactions of production/consumption (listed in Table S1), which are integrated into 11 governing equations (listed in Table S2). After building the biochemical network model and assessing the potential for bistability, we extend the conceptual scope of the system to include cytokeletal tension, resulting in 12 governing equations. The extended model with tension allows us to assess the influence of mechanical feedback on the qualitative behavior of the system.

## Results

Our first objective was to confirm that both directions of RhoA-NO mutual antagonism occur in the same cells. For this verification we selected an epithelial cell line (neither vascular nor highly contractile): MDCK cells stimulated with Hepatocyte Growth Factor (HGF).

### NO is required for growth factor-induced phosphorylation of RhoA

**Fig. 1:**
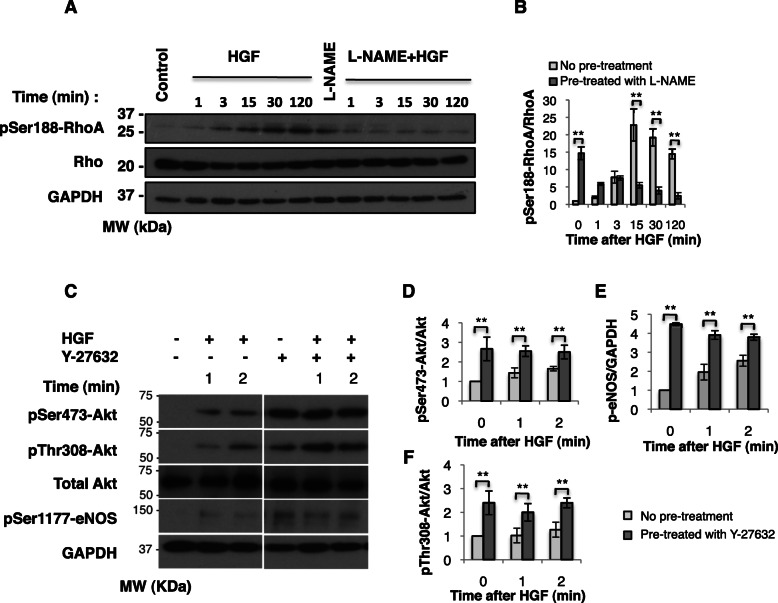
Experimental verification of mutual antagonism between NO and RhoA in epithelial (MDCK) cells. (A and B) NO mediates HGF-induced RhoA-Ser188 phosphorylation. MDCK cells were induced with HGF 1 ng/mL for the specified durations with or without a pre-treatment for 20 min with 50 μM L-NAME, an inhibitor of constitutive NOS. **A** Western blot analysis with pSer188-RhoA and **B** Quantification of band intensity. The phospho-RhoA-Ser188 bands in the western blot were quantified using ImageJ and normalized with respect to total RhoA intensity. **C**, **D**, **E** and **F** The Rho effector, ROCK inhibits p-Akt/p-eNOS signaling. MDCK cells were induced with HGF 1 ng/mL for the specified durations with or without 20 min pre-treatment with 20 uM of ROCK inhibitor Y-27632. **C** Western blot analysis for shorter durations of HGF treatment. Additional timepoints appear in Additional file 1. Commercial antibodies do not detect the canine isoform of eNOS but phospho-eNOS antibodies are reactive to the canine isoform. Quantification of the bands in the western blot: **D** phospho-Akt-Ser473 normalized against total Akt, **E** phospho-Akt-Thr308 normalized against total Akt, and **F** phospho-eNOS normalized against GAPDH. The white line between lanes denotes that a single gel was cropped to remove other treatment conditions in the middle lanes. (Full gel appears in Additional file 1). **p < 0.005

To test if NO antagonizes RhoA in epithelial cells, we selected serine-188 phosphorylation of RhoA as a proxy for the ability of NO to antagonize RhoA [25–27]. The effect of NO suppression on RhoA phosphorylation was measured in MDCK epithelial cells (Fig. 1A). RhoA is unphosphorylated in resting cells, and HGF triggers phosphorylation of RhoA at Serine 188. MDCK epithelial cells were treated with L-NAME, an inhibitor of cNOS, prior to treatment with HGF. We found that when NO was suppressed by L-NAME pre-treatment, then HGF stimulation was unable to phosphorylate RhoA at Serine 188. An additional side-effect of L-NAME was seen most clearly in cells without growth factor, where L-NAME treatment caused unexpected stimulation of phosphorylation. Such response has been observed in earlier studies where chronic administration of low dose of L-NAME suppressed the negative feedback regulation of NO synthesis by NO [37, 38] itself. For the purposes of confirming previously documented effects, in an epithelial cell type, we can conclude that NO was necessary for RhoA phosphorylation, suggesting that NO-induced antagonism of RhoA occurs significantly in this cell type.

### ROCK inhibits p-Akt/p-eNOS signaling

To test if the antagonistic effect of Rho/ROCK on eNOS is robust and extends to epithelial cells also, we examined whether the Serine 1177 phosphorylation of eNOS is regulated by RhoA/ROCK in MDCK cells. Western blot results (Fig. 1C) showed that treatment with ROCK inhibitor, Y-27632 increased Akt and eNOS phosphorylation. This effect was seen both after initial stimulation with HGF and in unstimulated cells. Additional file 1 Figure S8 (see Addition file 1) shows that long durations of HGF treatment induced an opposite effect, which might be due to a counter-balancing phosphatase [39]. In summary, inhibition of ROCK caused increased Akt and eNOS activation 1 to 2 min after HGF induction, indicating that ROCK inhibited phospho-Akt/phospho-eNOS signaling. We have established that both NO pathway inhibition of RhoA and RhoA pathway inhibition of NO can occur in MDCK cells.

### Relationship between mutual antagonism and Bistability

**Fig. 2:**
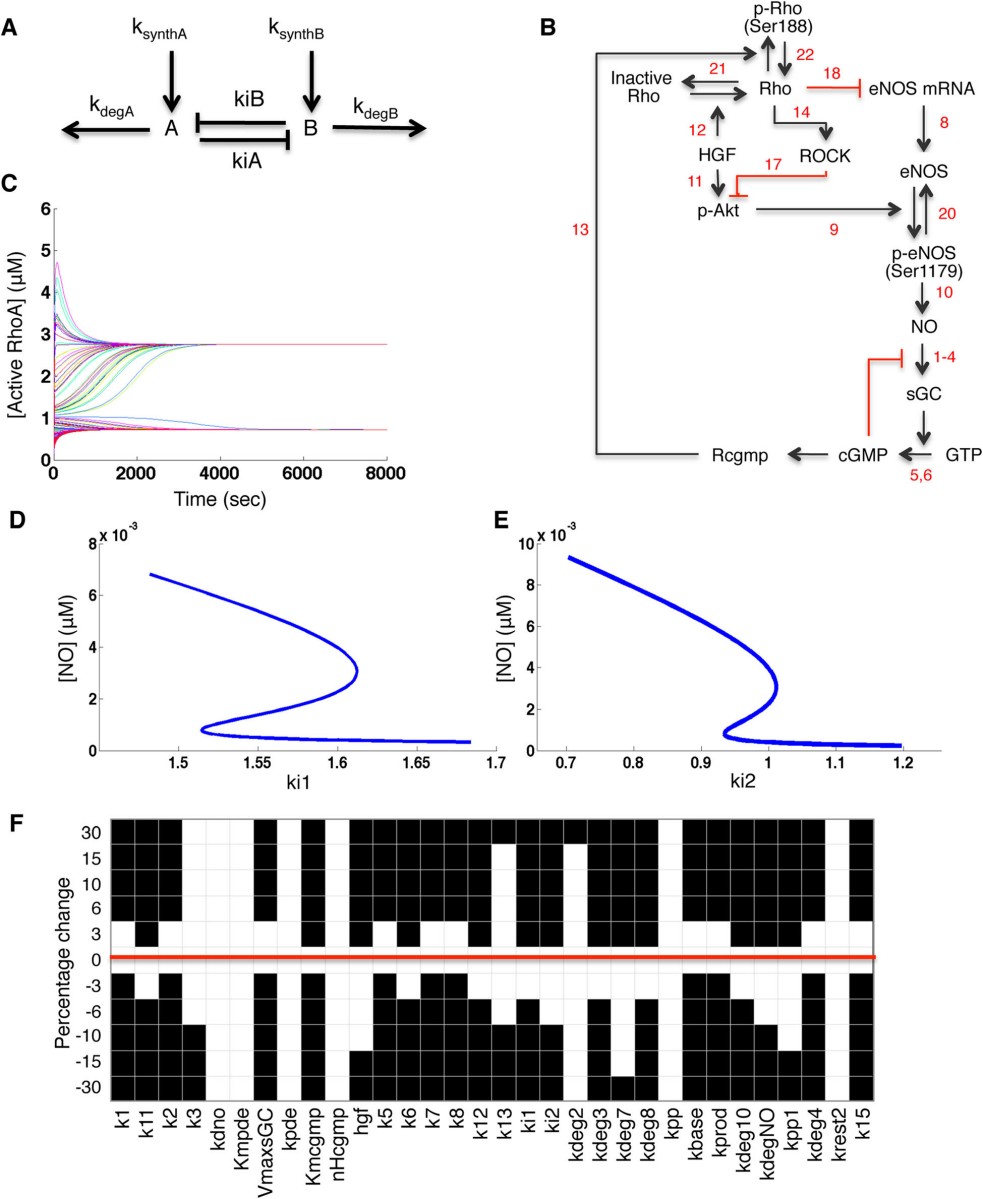
Network diagram and simulations of the initial model of HGF-activated RhoA-NO network, including known biochemical effects of mutual antagonism. **A** Two-node model to demonstrate that mutual antagonism is not sufficient to induce bistability. A and B are two species with zeroth order production and first order degradation rates, ksynthA, kdegA, ksynthB, kdegB for A and B respectively. A and B antagonize each other with rate constants kiA and kiB, according to the mass action equations in Additional file 1 Text S7. **B** The red arrows represent the inhibitory reactions. Complete specifications of the reactions appear in Additional file 1 Tables S1 and S2. **C** Model simulation with 100 random initial concentrations. The model was simulated, starting with random levels of all species. The resulting trajectories of the RhoA concentration over time (plotted with a random color for each trajectory) shows convergence to two different steady states. **D** and **E** Bifurcation analysis of the initial model. The steady state NO concentration is plotted against variation in the parameter **D** ki1 (rate constant for ROCK inhibition of Akt phosphorylation) and **E** ki2 (rate constant for RhoA inhibition of eNOS mRNA). **F** Robustness analysis of the bistability in the initial model. Single-variable robustness analysis was performed (see Procedure 4 in Additional file 1 Methods). Each of the parameters, from k1 to k15 was varied by the specified percentage, and the system was checked for bistability. White boxes denote the presence of bistability while black boxes denote the absence of bistability. The red line highlights the unperturbed parameter set. Note that bistability was retained in some columns, particularly for parameters involved in the degradation or inactivation of NO, eNOS, and cGMP

Mutual inhibition between two species is not sufficient by itself to induce bistability, To illustrate this, we simulated a minimal 2-species model of mutual antagonism (double-negative feedback) with mass action kinetics in Fig. 2A. Computational testing of rate parameters (using a multidimensional grid of possible combinations of values) showed that the system was monostable for all combinations (see Additional file 1, Additional file 1 Text S7). A double-negative system can exhibit bistability if one of the inhibitory reactions is ultrasensitive or if there are other forms of ultrasensitivity in the network [6], such as positive feedback. Therefore, the presence of mutual antagonism in the RhoA-NO system is not sufficient to indicate whether the system would be bistable.

### Pathway model of RhoA-NO mutual antagonism

To address whether the system is capable of bistability, we performed computational modeling of qualitative network behaviors. A signaling network model was constructed (Fig. 2B) using known biochemical effects and using a previous model of cGMP dynamics [40]. The model simulates changes in concentration over time. We applied a constant input of HGF stimulation to prevent the species from decaying to zero. The reactions in the model are described in Additional file 1 Text S3 (see Additional file 1). Although we simulate RhoA and NO concentrations in the physiological range, the model results should not be used for estimating absolute magnitudes; rather it will be used for generating testable predictions about qualitative behaviors. Even if each reaction could be isolated and quantified, the living cell has unknown effects from scaffolds and competitors.

### Bistability in the model

We searched the parameter space of the model for bistability (See Additional file 1, Additional file 1 Text S6, Procedure 2). Next we simulated the model using random initial configurations (See Additional file 1, Additional file 1 Text S6, Procedure 3), and found that the model consistently converged to one of two steady-state concentrations (Fig. 2C). The absolute magnitudes are not predictive, but the crucial observation is that the two states are significantly different from each other and that when in each state, the system remains at the same concentration indefinitely. This result implies that the system is capable of bistability.

To study the nature and extent of the bistable regime, we determined the steady-state(s) of the system for a range of values for the antagonism parameters ki1 and ki2. Figure 2D and E show the relationship between the parameter values and the NO steady-state levels. When the antagonism is very strong or very weak, the system has only one steady-state (monostable), but there exists an intermediate range for which the model has multiple steady-states. This is the characteristic S-shaped curve of bistability, which has two stable steady-states (bistable), plus an unstable steady-state that does not occur in practice. For further information about bifurcation diagrams and the fixed points of dynamical systems, please consult [7].

Next we studied whether bistability was robust to changes in the model parameters. Because this network is large and contains poorly studied reactions, robustness would be crucial for the biological plausibility of bistability. Single parameter perturbations (Fig. 2F) showed that for the majority of parameters, even a small percent deviation caused the system to lose bistability. We conclude that the bistability of this model is brittle, not robust. We are not aware of any systems that lacked robustness in theory but displayed robustness in experiments, so we infer that bistability would be an unlikely behavior for this system alone.

### Including mechanical tension in the model

**Fig. 3:**
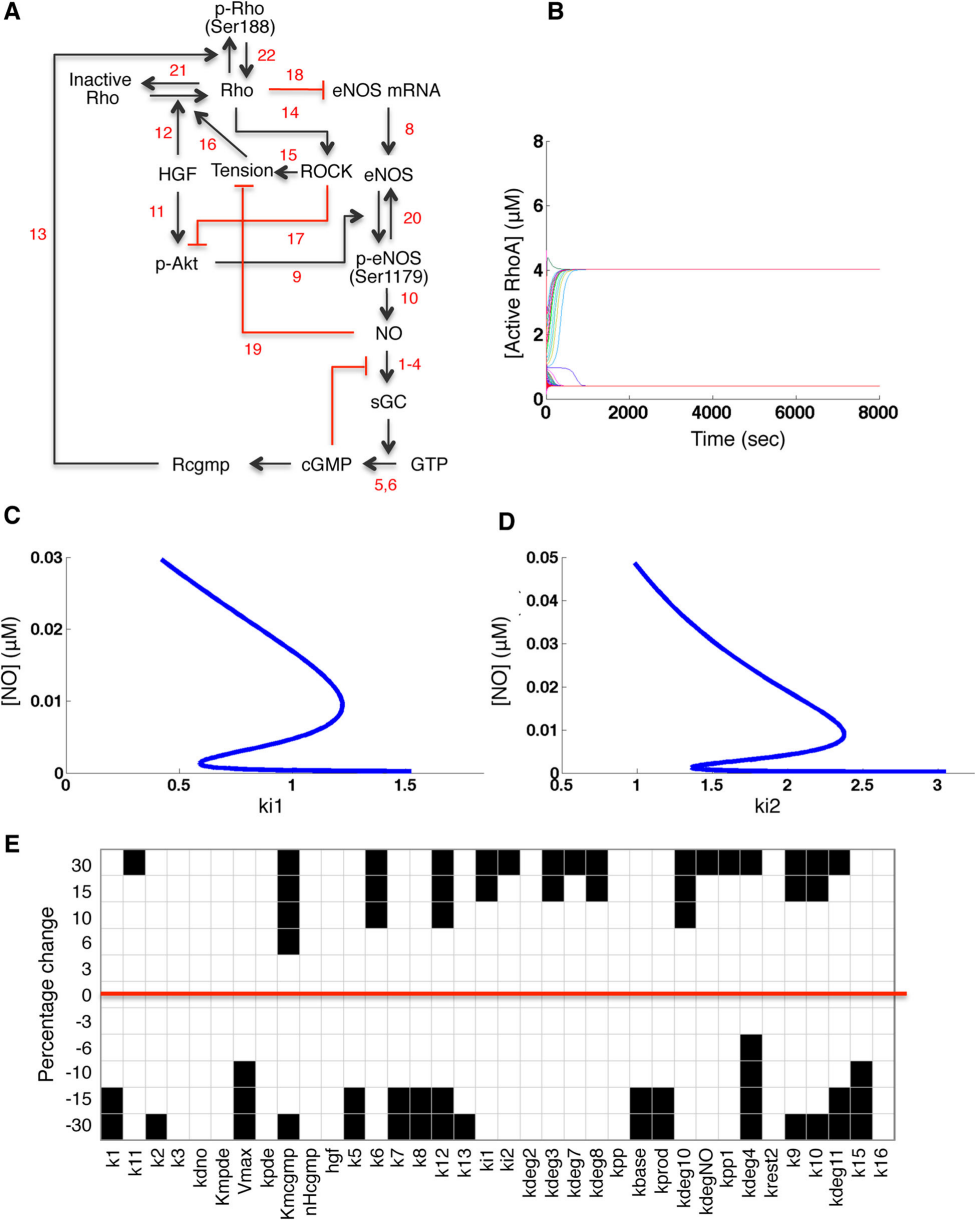
Network diagram and simulations of the extended model of HGF-activated RhoA-NO network, including known effects of ‘tension’ on NO and RhoA. **A** Red arrows represent the inhibitory reactions. Complete specification of the extended model appears in Additional file 1 Tables S1 and S2. **B** Simulation of the extended model, starting with 100 random levels of all species. Trajectories of the RhoA concentration show convergence to two different steady states. Note faster convergence than in Fig. 2. **C** and **D** Bifurcation analysis of the extended model. The steady state NO concentration is plotted against change in parameters **C** ki1 (rate constant for ROCK inhibition of Akt phosphorylation) and **D** ki2 (rate constant for RhoA inhibition of eNOS mRNA). Note that bistability occurs over a wider range of ki1 and ki2, compared with Fig. 2. **E** Robustness analysis (as in Fig. 2) applied to the extended model. White boxes denote the presence of bistability while black boxes denote the absence of bistability. The red line highlights the unperturbed parameter set

Thus far, we have neglected mechanical tension, but NO and RhoA have well-studied molecular mechanisms for exerting opposite effects on cytoskeletal tension. Indeed, their effects on tension are the reason we have studied both together. We next built an extended model (Fig. 3A) including a simple overall measure of cellular tension as if tension were a species in the reaction network. The NO/cGMP pathway has multiple well-studied mechanisms of decreasing cytoskeletal tension [25, 26, 41], which are represented by Reaction 19 in the extended model. Reaction 15 summarizes the mechanisms by which ROCK increases tension [42]. Finally we include the ability of mechanical tension to activate RhoA [43, 44] in Reaction 16, which is a form of positive feedback with ROCK activity [42]. Another potential source of positive feedback from ROCK to RhoA is mediated by phosphorylation of p190A-RhoGAP [45]. Rho-GEFs and Rho-GAPs are not included in this model, but the results we obtain about tension-mediated feedback are potentially relevant to p190A-RhoGAP feedback as well. Full specification of our reactions appears in Additional file 1 Text S3 (see Additional file 1). In summary, our extended model describes known cross-talk between the biochemical network and a first-order approximation of cellular tension.

We proceeded to test whether the extended model exhibited bistability. Figure 3B shows that the system converged stably to two different steady-states, RhoA-high and RhoA-low, depending on the initial concentrations given to the model. Like the purely biochemical model, the extended model was capable of bistability. Computing bifurcation diagrams for the extended model (Fig. 3C and D) showed that bistability was maintained over a wider range (five-fold increase) of the antagonism parameters. The difference in NO concentration between the two steady-states also increased 5-fold. Single parameter robustness analysis confirmed that the model was capable of bistability for a wider range of the parameters. The system retained bistabliity for 10 to 15% perturbation in 33 out of 36 parameters (Fig. 3E). The bistability was still sensitive to three parameters: Kmcgmp (the rate constant for cGMP in the Hill equation), k12 and kdeg4 (the rate constants for synthesis and degradation of ROCK). These three parameters belong to the “RhoA-side” of the model, suggesting that the effects of tension on RhoA, and generally the feedback loop between RhoA and tension, would be particularly important topics for future study, as they have disproportionate impact on the feasibility of bistability. Robustness is a relative term, and previous simulations of bistability in comparable biochemical signaling networks have shown several cases where 20 to 40% robustness in theory [1, 46, 47] led to cell culture experiments confirming bistability in practice. Because our system retained bistabliity for 30% perturbation in 23 out of 36 parameters, we conclude that bistability is credible enough to begin experimental testing.

## Discussion and conclusions

**Fig. 4:**
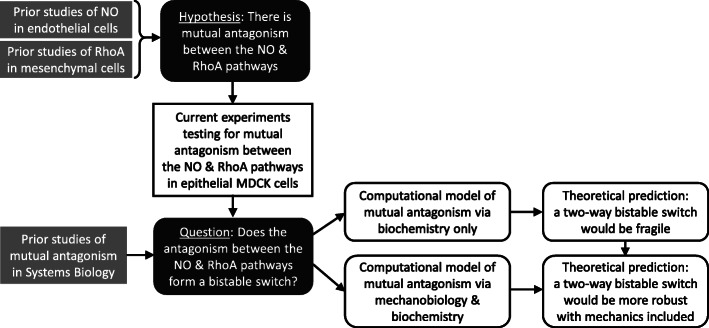
Flowchart of prior work and current work. Theoretical work appears in rounded boxes and experimental work appears in rectangles. Current contributions are in black type on white background. Guiding motivations are shaded black. Prior work is shaded grey

With the use of qualitative modeling, the primary outcome of our work is to assess whether the network topology is consistent with bistability and to find a range of parameter values and concentration levels that could cause bistability (see Additional file 1, Additional file 1 Table S1). Figure 4 shows the flow of logic for this study. An initial ‘tension-free’ model was found capable of bistability for NO levels ranging between 1 to 8 nM (Fig. 2D, E), but the bistability was easily disrupted by parameter perturbations. In the extended model with tension, the bistable regime could range from 1 to 30 nM of NO (Fig. 3C, D). This is within an order of magnitude of the 220 nM NO, previously measured in cerebellar cells [48]. Since the extended model exhibited bistability for moderate variation in most of the parameters, we conclude that the RhoA-NO bistability in silico is sufficiently robust to warrant the investment of testing for bistability in vitro.

Testing for bistability will be challenging because real-time sensors for RhoA activity can provide poor signal-to-noise ratio [49], and tools for dynamic detection of NO have many limitations, for example DAF has non-specificity [50], irreversible binding to NO, and photo-bleaching. Innovations in biosensor technology may enable these limitations to be overcome [51–53]. The size of the swing between two bistable states, and the ease of detecting the switch experimentally, would depend on the cell type. Based on our model, the concentration fold-change between high-NO and low-NO states would be highly dependent on parameters such as ki1 and ki2 (Fig. 3C, D) which would depend on cell type; endothelial cells would be expected to exhibit higher levels of NO in the high-NO steady-state, consistent with low values of ki1 and ki2 in Fig. 3C, D, while epithelial cells might be expected to have lower tolerance for NO, consistent with high values of ki1 and ki2.

Several important phenomena have been omitted from our present model. One is the ability of mechanical tension to regulate NO levels. Mechanosensors like the glycocalyx [54] mediate shear stress-induced NO production in endothelial cells. In addition, softening of the actin cortex may induce NO production [55]. If relaxation does have positive feedback, that could further increase the robustness of bistability. Another consideration is the effect of post-translational modification on protein stability. Ser188 phosphorylation of RhoA protects RhoA from degradation [32], so that NO/cGMP/PKG signaling can up-regulate RhoA protein expression, even though it down-regulates RhoA activity. For example, basal NO release from endothelial cells was found to be necessary for maintaining long-term RhoA levels in vascular smooth muscle cells [31]. Other post-translational effects we have omitted include phosphorylation of p190ARhoGAP [45], S-nitrosation of RhoA [56], and tyrosine nitration of RhoA [57] and p190Rho-GAP [58]. In the temporal realm, we have not yet studied dynamic stimuli such as pulsatile tension or oscillatory RhoA [49].

The importance of our work comes from the potential importance of bistability for converting noisy and gradual inputs into distinct transitions and regulatory decisions in the cell. Bistability may have physiological importance for the ability of cells to establish distinct spatial domains of effect, if nearby regions of space exhibit opposite steady states. In the cytoskeleton, bistability might sharpen the boundary between tension and relaxation, which would be useful for cell migration, direction sensing, and establishment of polarity, which are bistable decisions at the highest scale of cellular decision-making [11, 12]. Regional control of NO is reasonable, even though NO has fast diffusion, because NO exhibits narrow spatial targeting in vivo [59]. Bistability might explain how NO can be so narrowly confined, because there could be some regions having a self-reinforcing state of high NO production and other regions blocking NO production. A sharp concentration gradient would then occur between the two regions. A sharp concentration gradient can be interpreted as a spatiotemporal boundary for localizing tension within the cytosol.

In some examples of bistability, a “digital” yes/no decision at the molecular level might appear gradual or graded when viewed at a higher scale [8]. The assembly and disassembly of focal adhesions appears gradual, but it is oppositely regulated by RhoA and NO [60, 61], and is likely to be a discrete decision at the molecular scale [62–64]. Regions of greater mechanical tension would have greater positive feedback toward Rho activation, pushing them to a high-Rho state. The high-Rho state would promote stress fiber growth and focal adhesion assembly, consistent with observations of where adhesions form [65]. Regions with less tension would converge toward a high-NO state, and NO would promote disassembly of filamentous actin [66–68] and disassembly of adhesions. Because bistability aids in establishing sharp concentration gradients over space, RhoA and NO might have mutually exclusive regions of local up-regulation. Mutually exclusive regions of RhoA and NO could help explain the simultaneous assembly and disassembly of single focal adhesions, observed in heel-toe remodeling of adhesion structure [69].

In endothelial remodeling, shear stress triggers NO production through calcium-dependent pathways not included in our model [70], but the downstream effects of NO might still participate in switch-like behavior with RhoA. Blood flow creates localized regions of high force, inducing adhesions to grow at the upstream interface of the cell [71] with the activation of RhoA [72]. Meanwhile, the remainder of the cell (and its neighboring vascular smooth muscle cells) might relax in response to the shear-induced NO. RhoA-NO bistability might facilitate an orderly juxtapositioning of adhesion and relaxation that benefits cell health, in addition to the known importance of laminar flow versus turbulent flow. There are many possible contexts where tension-dependent bistability between RhoA and NO could contribute to the self-organization of cytoskeletal machinery into discrete spatiotemporal domains.

## Materials and methods

### Cell culture experiments

Madin-Darby Canine Kidney (MDCK) strain II cells were kindly provided by Walter Hunziker from IMCB-Singapore [73]. Chemicals (L-NAME (L-NG-Nitroarginine Methyl Ester), Y-27362, HGF) were from Sigma. Additional file 1 Texts S4 and S5 (see Additional file 1) describe cell culture methods, sample preparation, Western blotting method and antibody reagents.

### Computational procedures and bifurcation analysis

The differential equations were simulated in Matlab (Mathworks, Natick, MA). Procedures for assessing bistability, for parameter search, for random generation of 100 initial conditions, and for robustness analysis appear in Additional file 1 Text S6 (see Additional file 1). Similar methods have been used previously [74, 75]. For bifurcation diagrams, we computed the change in steady-states over a range of parameters, using Govaerts and Kuznetsov’s continuation software Matcont [76], and Matcont parameters were chosen to ensure the bistable region was evaluated.

### Statistical analysis

Experiments were performed in triplicate. Error bars display the standard errors of the mean (±SEM). Statistical significance was determined using Student’s t-test.

## Supplementary Information

**Additional file 1.** Supplementary materials for “A computational model of mutual antagonism in the mechano-signaling network of RhoA and Nitric Oxide” including Table S1 (Reactions, parameters and variables for the initial and the extended models), Table S2 (Ordinary Differential Equations and the corresponding reactions from Table S1), Table S3 (Initial concentrations for extreme initialization of ‘NO-high’ & ‘RhoA-high’ states), Table S4 (Ordinary Differential Equations (ODEs) and parameter values), Texts S3, S4, S5, S6, S7 (Supplementary Materials and Methods), Figure S8 (Raw, unedited gel images of the western blot in Fig. 1A), Figure S9 (Raw, unedited gel images of the western blot in Fig. 1C) and Figure S10. (ROCK inhibits Akt/eNOS phosphorylation at shorter durations of HGF treatment while long durations of HGF treatment induced an opposite effect.)

## Data Availability

Not applicable.

## Abbreviations

NO: Nitric Oxide
ROCK: Rho Kinase
iNOS: Inducible Nitric Oxide Synthase
eNOS: Endothelial Nitric Oxide Synthase
cNOS: Constitutional Nitric Oxide Synthase
HGF: Hepatocyte Growth Factor
MDCK: Madin-Darby Canine Kidney cells
L-NAME: L-N-Nitro arginine methyl ester
RhoGAP: Rho GTPase-activating protein
GEF: Guanine nucleotide exchange factor
