# Supplementary material for: A computational model of mutual antagonism in the mechano-signaling network of RhoA and nitric oxide
Source: BMC Mol Cell Biol. 2021 Oct 12;22(Suppl 1):47. doi: 10.1186/s12860-021-00383-5 (PMC8507106; doi:10.1186/s12860-021-00383-5)
Supplement: Supplementary file 1 — Additional file 1. Supplementary materials for “A computational model of mutual antagonism in the mechano-signaling network of RhoA and Nitric Oxide” including Table S1 (Reactions, parameters and variables for the initial and the extended models), Table S2 (Ordinary Differential Equations and the corresponding reactions from Table S1), Table S3 (Initial concentrations for extreme initialization of ‘NO-high’ & ‘RhoA-high’ states), Table S4 (Ordinary Differential Equations (ODEs) and parameter values), Texts S3, S4, S5, S6, S7 (Supplementary Materials and Methods), Figure S8 (Raw, unedited gel images of the western blot in Fig. 1A), Figure S9 (Raw, unedited gel images of the western blot in Fig. 1C) and Figure S10. (ROCK inhibits Akt/eNOS phosphorylation at shorter durations of HGF treatment while long durations of HGF treatment induced an opposite effect.) [file 12860_2021_383_MOESM1_ESM.pdf]

Bistability and its Implications for Mechano-signaling: A Computational Model of Mutual Antagonism between RhoA and Nitric Oxide Signaling

Akila Surendran, C.Forbes Dewey Jr., Low Boon Chuan, Lisa Tucker-Kellogg

Supplementary material: 2 Tables, 5 Texts, and 3 Figure.

Supplementary tables

Table S1. Reactions, parameters and variables for the initial and the extended models.

	Reactions	Parameters and variables for 'Tension-free model'	Parameters and variables for 'Extended model'
1	$E_b + NO \underset{k11}{\overset{k1}{\rightleftarrows}} E_{6c}$	k1=2000 $\mu M^{-1} s^{-1}$<br>k11=100 s^{-1}	k1=2000 $\mu M^{-1} s^{-1}$<br>k11=100 s^{-1}
2	$E_{6c} \xrightarrow{k2} E_{5c}$	k2=0.1 s^{-1}<br>$E_{5c} = (1-E_b-E_{6c})\ \mu M$	k2=0.1 s^{-1}<br>$E_{5c} = (1-E_b-E_{6c})\ \mu M$
3	$E_{6c} + NO \xrightarrow{k3} E_{5c} + NO$	k3=3 $\mu M^{-1} s^{-1}$	k3=3 $\mu M^{-1} s^{-1}$
4	$E_{5c} \xrightarrow{k4} E_b + NO$	$k_4 = 0.098*(cGMP^m)$<br>m=1	$k_4 = 0.098*(cGMP^m)$<br>m=1
5	$GTP+E_{5c} \longleftrightarrow GTP.E_{5c} \longrightarrow cGMP + E_{5c}$	V_p= VmaxsGC * E_{5c}<br>VmaxsGC=1.09 $\mu M\ s^{-1}$	V_p= VmaxsGC * E_{5c}<br>VmaxsGC=1.09 $\mu M\ s^{-1}$
6	$cGMP + PDE \longleftrightarrow cGMP.PDE \longrightarrow GMP+PDE$	$V_d = \frac{cGMP*V\max pde}{Kmpde+cGMP}$<br>Vmaxpde = kpde*cGMP<br>Kmpde=2 μM<br>kpde=0.032 s^{-1}	$V_d = \frac{cGMP*V\max pde}{Kmpde+cGMP}$<br>Vmaxpde = kpde*cGMP<br>Kmpde=2 μM<br>kpde=0.032 s^{-1}
7	$NO \xrightarrow{kdno}$	kdno=0.01 s^{-1}	kdno=0.01 s^{-1}
8	$eNOSmrna \xrightarrow{kprod} eNOS$	kprod=0.5 s^{-1}	kprod=0.5 s^{-1}
9	$eNOS + pAkt \xrightarrow{k7} peNOS$	k7=0.1 $\mu M^{-1} s^{-1}$	k7=0.1 $\mu M^{-1} s^{-1}$
10	$peNOS \xrightarrow{k8} NO$	k8=0.4 s^{-1}	k8=0.8 s^{-1}

11	Akt $\xrightarrow[HGF]{k5}$ pAkt	k5=2 μM^{-1} s^{-1}<br>HGF=0.01 μM<br>Akt = (2.5 – pAkt) μM	k5=2.5 μM^{-1} s^{-1}<br>HGF=0.01 μM<br>Akt = (2.5 – pAkt) μM
12	Rho_inact $\xrightarrow[HGF]{k6}$ Rho_act	k6=2 μM^{-1} s^{-1}	k6=2 μM^{-1} s^{-1}
13	Rho_act $\xrightarrow[Rcgmp]{k15}$ pRho	k15=0.91 s^{-1}<br>Kmcgmp=0.55 μM<br>nHcgmp=2<br>*Rcgmp=* $\frac{cGMP^{nHcgmp}}{cGMP^{nHcgmp}+Kmcgmp^{nHcgmp}}$<br>pRho = (8-Rho_act-Rho_inact) μM	k15=1 μM^{-1} s^{-1}<br>Kmcgmp=0.55 μM<br>nHcgmp=2<br>*Rcgmp=* $\frac{cGMP^{nHcgmp}}{cGMP^{nHcgmp}+Kmcgmp^{nHcgmp}}$<br>pRho = (8-Rho_act-Rho_inact) μM
14	Rho_act $\xrightarrow{k12}$ ROCK	k12=1 s^{-1}	k12=1 s^{-1}
15	ROCK $\xrightarrow{k9}$ tension	-	k9=0.2 s^{-1}
16	Rho_inact + tension $\xrightarrow{k10}$ Rho_act	-	k10=0.07 μM^{-1} s^{-1}
Inhibition			
17	ROCK $\overset{ki1}{\dashv}$ pAkt	ki1=1.6 μM^{-1} s^{-1}	ki1=2.1 μM^{-1} s^{-1}
18	Rho_act $\overset{ki2}{\dashv}$ eNOSmrna	ki2=1 μM^{-1} s^{-1}	ki2=1
19	NO $\overset{k16}{\dashv}$ tension	-	k16=0.76 μM^{-1} s^{-1}
20	peNOS $\xrightarrow{kpp}$ eNOS	kpp=0.01 s^{-1}	kpp=0.01 s^{-1}
21	Rho_act $\xrightarrow{k13}$ Rho_inact	k13=0.01 s^{-1}	k13=0.1 s^{-1}
22	pRho $\xrightarrow{krest2}$ Rho_inact	krest2=1 s^{-1}	krest2=1 s^{-1}
Production			
23	$\xrightarrow{kbase}$ eNOSmrna	kbase=5 μM s^{-1}	kbase=5 μM s^{-1}
Degradation			
24	cGMP $\xrightarrow{k\deg 10}$	kdeg10=0.9 s^{-1}	kdeg10=0.9 s^{-1}
25	NO $\xrightarrow{k\deg NO}$	kdegNO=0.9 s^{-1}	kdegNO=0.9 s^{-1}

26	eNOSmrna $\xrightarrow{k\deg 7}$	kdeg7=0.5 s^{-1}	kdeg7=0.5 s^{-1}
27	eNOS $\xrightarrow{k\deg 8}$	kdeg8=0.99 s^{-1}	kdeg8=0.99 s^{-1}
28	peNOS $\xrightarrow{k\deg 3}$	kdeg3=0.9 s^{-1}	kdeg3=0.9 s^{-1}
29	pAkt $\xrightarrow{k\deg 2}$	kdeg2=0.05 s^{-1}	kdeg2=0.1 s^{-1}
30	ROCK $\xrightarrow{k\deg 4}$	kdeg4=0.99 s^{-1}	kdeg4=0.99 s^{-1}
31	Tension $\xrightarrow{k\deg 11}$	-	kdeg11=0.5 s^{-1}

Table S2. Ordinary Differential Equations (ODEs) and the corresponding reactions from Table S1.

Rate equation	From reactions numbered in **Table S1**
1. $\frac{d}{dt}[E_b]= -(k_1*E_b*NO) + (k_{11}*E_{6c}) + (k_4*E_{5c})$	1,4
2. $\frac{d}{dt}[E_{6c}] = (k_1*E_b*NO) - (k_{11}*E_{6c}) - (k_2*E_{6c}) - (k_3*E_{6c}*NO)$	1,2,3
3. $\frac{d}{dt}[cGMP] = V_p - V_d - (kdeg10*cGMP)$	5,6,24
4. $\frac{d}{dt}$[NO]=(k8*peNOS)-(kdno*NO)-(kdegNO*NO)	7,10,25
5. $\frac{d}{dt}$[eNOSmrna]=kbase-(ki2*Rho_act*eNOSmrna)-(kdeg7*eNOSmrna)	18,23,26
6. $\frac{d}{dt}$[eNOS]=(kprod*eNOSmrna)-(kdeg8*eNOS)-(k7*pAkt*eNOS)+(kpp*peNOS)	8,9,20,27

7. $\frac{d}{dt}$[peNOS]=(k7*pAkt*eNOS)-(kdeg3*peNOS)-(kpp*peNOS)	9,20,28
8. $\frac{d}{dt}$[pAkt]= (k5*hgf*Akt)-(ki1*ROCK*pAkt)-(kdeg2*pAkt)	11,17,29
9. $\frac{d}{dt}$(Rho_act)=(k6*Rho_inact*hgf)-(k15*Rcgmp*Rho_act)-(k13*Rho_act)+(k10*tension*Rho_inact)	12,13,16,21
10. $\frac{d}{dt}$(Rho_inact)=-(k6*Rho_inact*hgf)+(k13*Rho_act)-(k10*tension*Rho_inact)+(krest2*pRho)	12,16,21,22
11. $\frac{d}{dt}$(ROCK)=(k12*Rho_act)-(kdeg4*ROCK)	14,15,17,30
12. $\frac{d}{dt}$(tension)=(k9*ROCK)-(kdeg11*tension)-(k16*NO*tension)	15,16,19,31

Supplementary Texts

Text S3. Model construction

The reaction network for the initial network (**Figure 2B** and Supplementary **Table S1)** was constructed as follows. We describe the effect of NO on cGMP mediated by the activation of soluble guanylate cyclase (sGC) using a previous model by Yang et al. [1]. Their reactions and parameters appear in our model (Reactions 1 to 7). For sGC signaling, we adopted the reactions of Yang et al. [1], in which the basal, intermediate and fully activated forms of sGC are called E_b, E_{6c} and E_{5c} respectively. Regulatory effects of cGMP are described by the Rcgmp term [1] and we adapted their Hill equation to describe PKG kinase activity on RhoA, downstream of cGMP (Reaction 13). A key difference is that Yang et al. assumed NO would be a **constant input** of 220 nM, while

in our model, we simulate NO dynamics due to time-varying expression and phosphorylation of eNOS.

In our model, eNOS (produced by Reaction 8) is activated by phosphorylation, which is induced by p-Akt [2] (Reaction 9). Phosphorylated eNOS causes NO production (Reaction 10). Phosphorylation and activation of Akt to p-Akt is induced by HGF [2] (Reaction 11). At the same time, HGF induces activation of RhoA (Reaction 12) [3]. RhoA can be phosphorylated at Ser188 by cGMP/PKG signaling (Reaction 13) which depletes active RhoA [4, 5]. When RhoA is active, it activates Rho kinase, called ROCK (Reaction 14). We have simplified the RhoA-mediated conversion of inactive ROCK to active ROCK as generic production of active ROCK by RhoA. Next, ROCK inhibits Akt phosphorylation [6] (Reaction 17). Active RhoA inhibits eNOS mRNA stability [7], which is represented using a RhoA-dependent negative rate constant in the production of eNOS mRNA (Reaction 18).

A constant production of eNOS mRNA (Reaction 23) and a constant input of HGF were provided as stimulus to the system, so the activation levels would not decay to zero. The total concentrations of RhoA, Akt and sGC are conserved in the system as represented in the parameter values of pRho, Akt and E_{5c} in Reactions 13,11 and 2 respectively. All non-conserved species were given degradation rates (Reactions 24 to 31). To simplify the modeling of ROCK and Akt, their degradation terms also encompass inactivation and dephosphorylation. Inactivation was treated explicitly for eNOS and Rho (Reactions 20-22). Reactions 1-7 have fixed rates [1] and the remaining rates were adjusted to achieve bistability (see Supplementary text S6, Procedure 2).

For the 'Extended model' (**Figure 3A**), we added three new reactions to the 'Initial' model. In the first new reaction (Reaction 15), ROCK increases tension because the RhoA effector ROCK increases myosin phosphorylation and myosin motor function

directly [8], and also because it acts indirectly by phosphorylating and inactivating myosin phosphatase [9]. In the second new reaction (Reaction 16), tension causes greater activity of RhoA because membrane tension causes activation of RhoA in multiple systems [10-12] and matrix rigidity causes increased activation of RhoA [10]. In the third new reaction (Reaction 19), NO decreases tension. NO is a well-studied trigger for vasorelaxation [13-15], and some of the ways it inhibits cytoskeletal tension and myosin motor activity are by decreasing intracellular Ca^{2+} levels [15], activating K^+ channels [16] and activating myosin phosphatase [17].

Text S4. Cell Culture

Madin-Darby Canine Kidney (MDCK) strain II cells were grown in high-glucose Dulbecco's modified Eagle medium (DMEM, Hyclone) supplemented with 10% (v/v) fetal bovine serum. The cells were grown at 37°C with 5% CO_2. Cells were passaged every other day with passage ratio 1:5.

Text S5. Sample preparation and Western blotting

Cells were lysed using RIPA buffer (150 mM sodium chloride, 50 mM Tris-HCl pH 7.3, 0.25 mM EDTA, 1% (w/v) sodium deoxycholate, 1% (v/v) Triton X-100, 50 mM sodium fluoride, 5 mM sodium orthovanadate, and protease inhibitors (Roche Applied Science)). The lysates were then centrifuged for 10 minutes in 4°C at 12000 rpm. For western blotting analysis the supernatant was mixed with SDS loading dye and boiled at 85°C for 5 minutes. Proteins were separated by SDS-PAGE and transferred to a PVDF membrane (Millipore). The PVDF membrane with transferred protein was incubated in blocking buffer containing 10 ml of 1% BSA in 1x PBS with 0.1% tween-20 (PBST) at room temperature with gentle shaking for 1 hour. The blocking solution was replaced by

the primary antibody with appropriate dilution prepared in 1% BSA in 1x PBST and shaken overnight at 4ºC. After incubation, the membrane was washed thrice with PBST for 5 minutes each on a shaker. The matching secondary antibody conjugated with HRP with 1:2500 dilution in 1% BSA in PBST was added to the membrane and shaken gently at room temperature for 1 hour. This was followed by five washes with PBST for 5 minutes each. After immunoblotting, the HRP activity was detected by enhanced chemiluminescent substrate (Pierce Biotechnology, Inc.). Rabbit polyclonal antibodies anti-pSer1177-eNOS, anti-pSer473-Akt and anti-pThr308-Akt were from Cell Signaling; anti-pSer188-RhoA was from Sigma. Mouse monoclonal antibodies anti-Akt was from BD Transduction Laboratories; anti-GAPDH and anti-RhoA were from Sigma.

Text S6. Procedures for computational modeling of bistability

Procedure 1. Test for bistability and Determination of D^{ss}

If a system is bistable, it can converge toward either of two stable steady-states, depending on the initial conditions. Because the scope of our system is defined by the mutual antagonism between NO and RhoA, we assume that multiple steady states, if they exist, would have different levels of NO and RhoA. To test for convergence to multiple steady states, we initialized the system once with high levels of NO and once with high levels of RhoA, before observing steady state convergence. If the system converges to different steady states, depending on the initial concentrations, then the system is not monostable.

1. To initialize the system with high NO, the species eNOS mRNA, phospho-Akt and phospho-RhoA were set to extreme, non-physiological concentrations (see table

below). To initialize the system with high RhoA, the species active RhoA, ROCK and tension were set to high concentrations.

Extreme values are used because initializing the system with very high or very low levels serves to demonstrate a mathematical property of the system. If a system is initialized at points near the steady state, then convergence to the steady state only serves to establish a small basin of attraction around the steady state. In contrast, if the system is initialized very far from the steady state, and if the system still succeeds at converging to the steady state, this convergence indicates a larger basin of attraction for the steady state. Convergence from a distant point necessarily passes through nearer points before arriving at the final state, so in mathematical terms, the extreme experiment contains the smaller experiment as a subset of its trajectory. A more physiological experiment would have a less extreme initialization, but the more physiological experiment is technically a subset of what we demonstrate when we perform the extreme initialization.

Initial concentrations for extreme initialization of 'NO-high' & 'RhoA-high' states

Species	Initial concentration for NO-high state (μM)	Initial concentration for RhoA-high state (μM)
E_b	1	1
eNOS mRNA	100000	5
Active RhoA	0	8
pSer188-RhoA	8	0
p-Akt	100000	0
ROCK	0	100000
Tension	0	100000

Initial concentration of HGF was 0.01 μM and that of all the other species were 0 μM.

2. For each set of initial concentrations (NO-high or RhoA-high), the ODE equations were solved (computing the trajectories of the species over time) for 2,000,000 steps, which was sufficient for all models to converge to steady state. Steady state is the condition where there is no further change in concentrations with respect to time.
3. The steady state concentration of RhoA after initialization with the NO-high conditions ($SS\text{-}NO^{high}$), and the steady state concentration of RhoA after initialization with the RhoA-high conditions ($SS\text{-}Rho^{high}$) were used to compute the difference between RhoA steady states, $D_{ss} = SS\text{-}Rho^{high} - SS\text{-}NO^{high}$. Note that if RhoA levels differ, then most other species of the system would differ as well, but we chose RhoA to represent the difference in steady states.
4. If the absolute value of D_{ss} was greater than 0.0001, the system was considered bistable (or multistable), otherwise it was considered to be monostable.

Procedure 2: Search parameter space for bistability

We searched for parameters that balanced the antagonism between NO and RhoA as follows. First the parameters ki1 (the rate of ROCK antagonizing phospho-Akt) and k15 (the rate of cGMP-mediated phosphorylation of RhoA at Ser 188) were adjusted to ensure D_{ss} would be non-negative. (A negative value of D_{ss} means that initializing the system with higher RhoA leads to a steady state with lower RhoA, indicating poor locality of the convergence behavior, which would be an undesirable property for the model.) Next, an automated search was performed, seeking combinations of rate parameters causing D_{ss} to be positive. This was followed by a final phase of manual parameter tuning to increase the absolute value of D_{ss}.

Procedure 3: Random generation of 100 initial conditions

1. For a given bistable model, the stable steady states were determined using Procedure 1. Let vectors $\mathbf{x}_1$ and $\mathbf{x}_2$ denote the species concentrations of the two stable steady-states.

2. Define $\mathbf{x}_m$ to be the mid-point between $\mathbf{x}_1$ and $\mathbf{x}_2$.

3. 100 species vectors $\mathbf{x}_i$ (for i=1..100) were generated from the Gaussian distribution with mean $\mathbf{x}_m$ and standard deviation 0.25*($\mathbf{x}_2$-$\mathbf{x}_1$).

4. Starting from each of the $\mathbf{x}_i$ as initial concentrations, the model was simulated for 2 million steps, which was sufficient for to achieve steady state in all cases.

Procedure 4: Robustness analysis by single parameter perturbation

1. The robustness of bistability to each rate parameter was determined by varying single parameters individually.

2. Each parameter was increased or decreased by 3%, 6%, 10%, 15%, or 30%, while holding all other rate parameters fixed to their nominal values (Table S1).

3. For each parameter and each percent increase or decrease, bistability was evaluated using Procedure 1.

Text S7. Search for Bistability in Two-Node Model of Mutual Antagonism.

A two-node mass action model (**Figure 2A)** consisting of two species was constructed to demonstrate that mutual antagonism is not sufficient to induce bistability. A and B are two species with zeroth order production rates k_{synthA} and k_{synthB} respectively. The first order degradation rates of A and B are kdegA and kdegB respectively. A antagonizes B, which is represented by a second-order A-dependent reaction that consumes B with rate

constant kiA. In other words, the term kiA * [A] * [B] appears as a negative entry in the differential equation for [B] over time. A symmetric relationship was added for B to antagonize A with rate constant kiB.

The model was simulated many times with different combinations of parameter values. The parameters values were taken from a 4-dimensional grid of possible values for kdegA, kdegB, kiA, and kiB, as listed in the table below. Each parameterization of the model was tested for bistability following a procedure similar to **Procedure 1** above**.** The extreme initialization of concentration used here for the 'A-high' state was:

[A] = 10000 µM and [B] = 0 µM.

For the 'A-low' state we used [A] = 0 µM and [B] = 10000 µM.

For each parameterization, the model converged to the same steady state, regardless whether it was initialized with A-high or A-low. The absence of bistability in the two-node mass action model is a concrete demonstration of the general principle that bistability requires ultrasensitivity [18].

Rate equation	Parameter values or range of parameter values used to test bistability (initial value: step size: final value)
1. Reaction for A inhibiting B: $\frac{d}{dt}$[A] = k_{synthA}-(kiA*B*A)-(kdegA*A)	k_{synthA}=5<br>kiA =1:1:20<br>kdegA = 0.1:0.1:0.9
2. Reaction for B inhibiting A: $\frac{d}{dt}$[B] = k_{synthB}-(kiB*A*B)-(kdegB*B)	k_{synthB}=5<br>kiB = 1:1:20<br>kdegB = 0.1:0.1:0.9

A

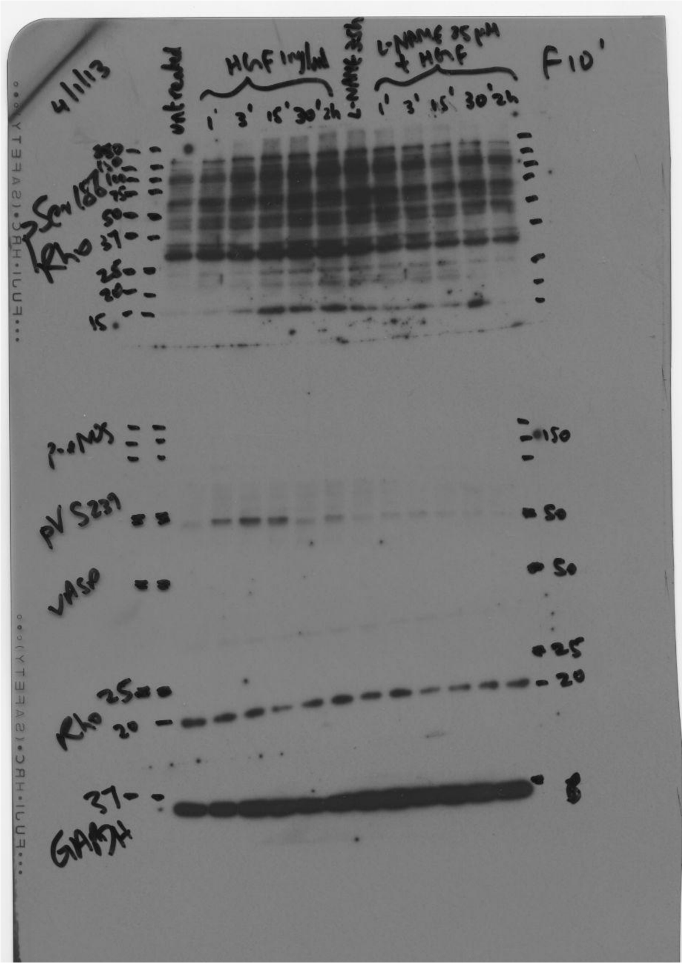

B

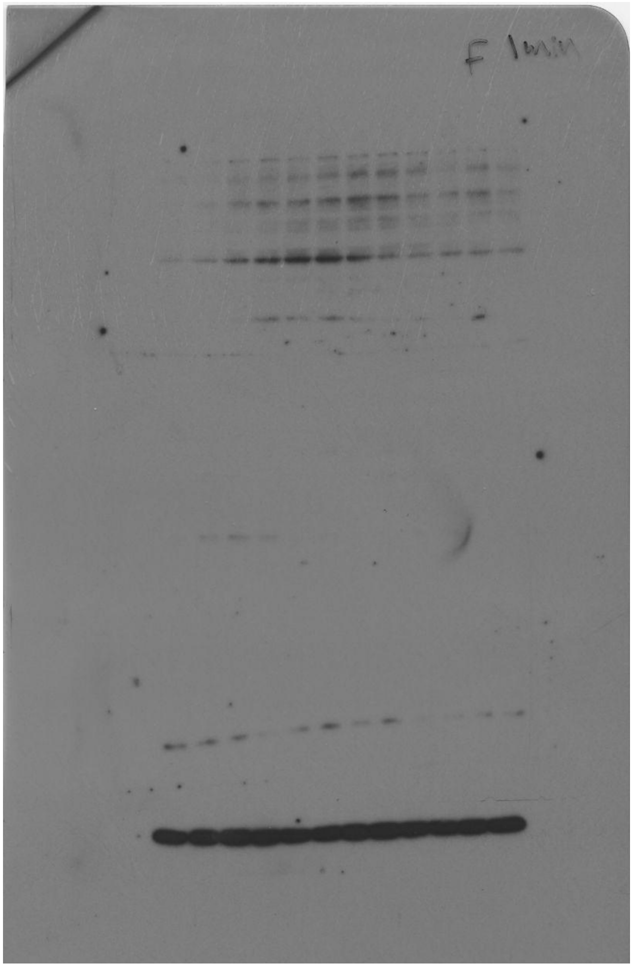

C

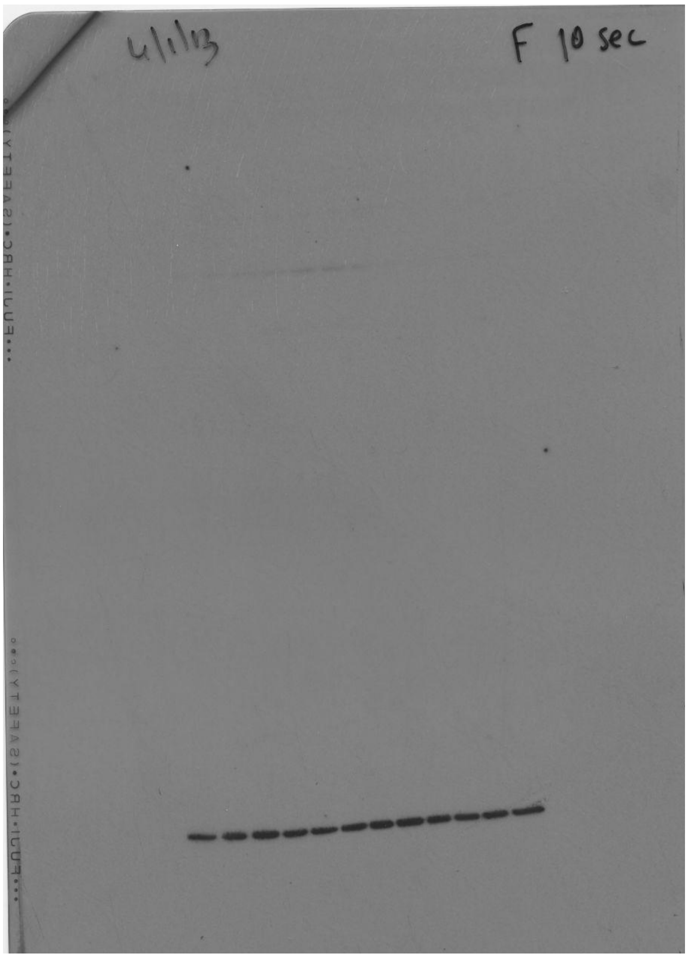

Figure S8: Raw, unedited gel images of the western blot in Figure 1A. (A) Slow exposure for ten minutes (B) Medium exposure for one minute (C) Quick exposure for 10 seconds

A

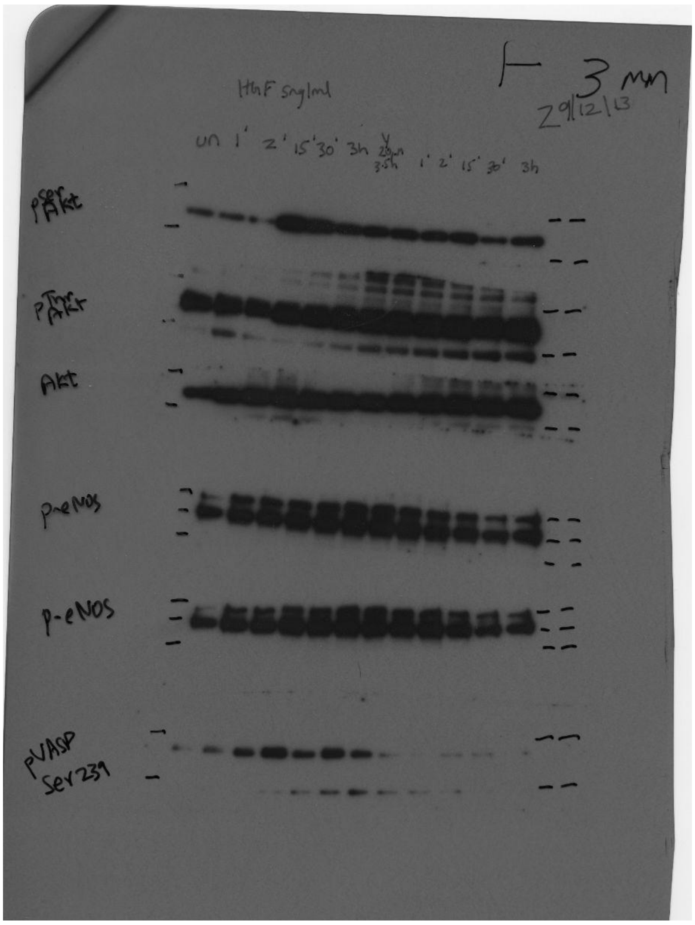

B

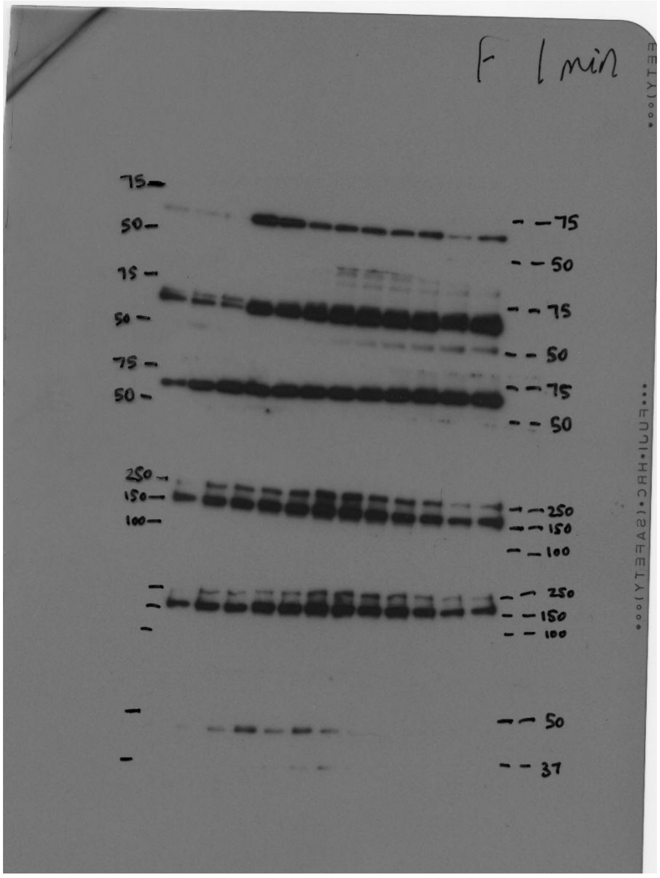

C

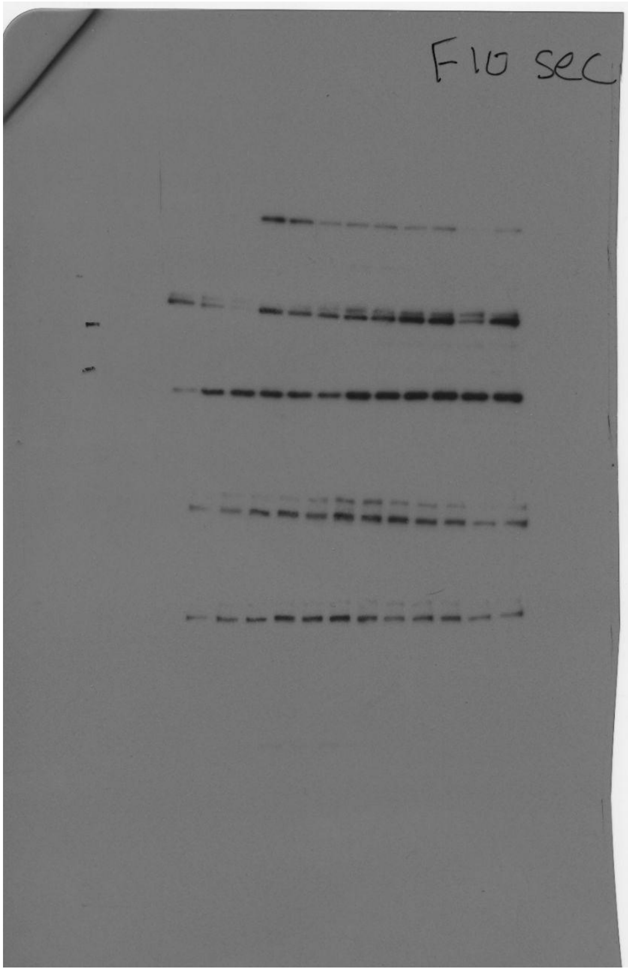

Figure S9: Raw, unedited gel images of the western blot in Figure 1C. (A) Slow exposure for three minutes (B) Medium exposure for one minute (C) Quick exposure for 10 seconds

Figure S10 ROCK inhibits Akt/eNOS phosphorylation at shorter durations of HGF treatment while long durations of HGF treatment induced an opposite effect. *MDCK cells were induced with HGF 1ng/mL for the specified durations with or without 20 minute pre-treatment with 20 uM of ROCK inhibitor Y-27632.*

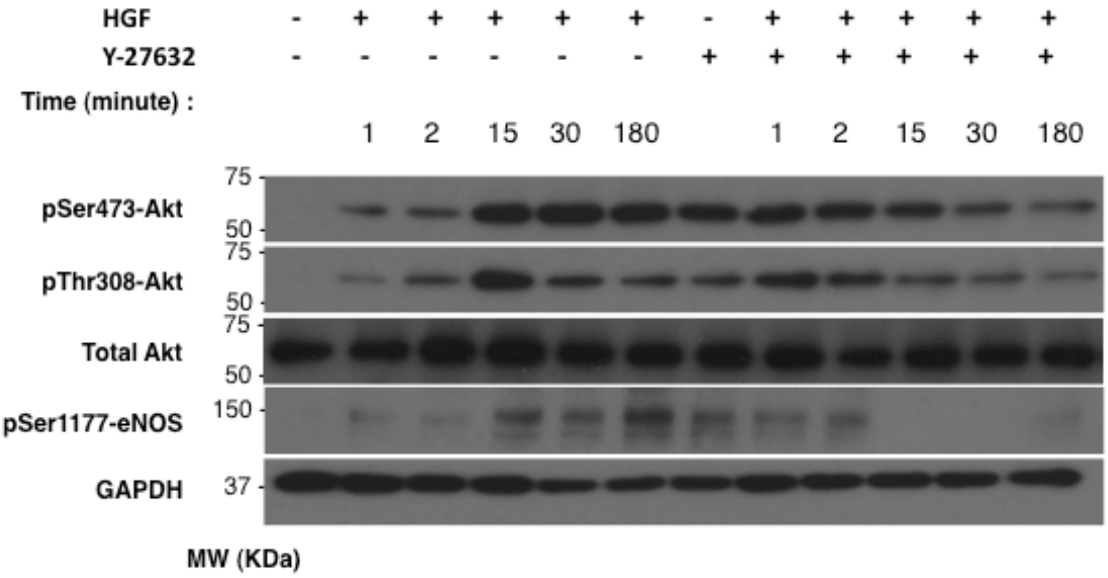

References for Supplementary Materials S1-S8

1. Yang, J., et al., *Mathematical modeling of the nitric oxide/cGMP pathway in the vascular smooth muscle cell.* Am J Physiol Heart Circ Physiol, 2005. **289**(2): p. H886-97.
2. Uruno, A., et al., *Hepatocyte growth factor stimulates nitric oxide production through endothelial nitric oxide synthase activation by the phosphoinositide 3-kinase/Akt pathway and possibly by mitogen-activated protein kinase kinase in vascular endothelial cells.* Hypertens Res, 2004. **27**(11): p. 887-95.
3. Royal, I., et al., *Activation of cdc42, rac, PAK, and rho-kinase in response to hepatocyte growth factor differentially regulates epithelial cell colony spreading and dissociation.* Mol Biol Cell, 2000. **11**(5): p. 1709-25.

4. Rolli-Derkinderen, M., et al., *Phosphorylation of serine 188 protects RhoA from ubiquitin/proteasome-mediated degradation in vascular smooth muscle cells.* Circ Res, 2005. **96**(11): p. 1152-60.
5. Sauzeau, V., et al., *RhoA expression is controlled by nitric oxide through cGMP-dependent protein kinase activation.* J Biol Chem, 2003. **278**(11): p. 9472-80.
6. Ming, X.F., et al., *Rho GTPase/Rho kinase negatively regulates endothelial nitric oxide synthase phosphorylation through the inhibition of protein kinase B/Akt in human endothelial cells.* Mol Cell Biol, 2002. **22**(24): p. 8467-77.
7. Laufs, U. and J.K. Liao, *Post-transcriptional regulation of endothelial nitric oxide synthase mRNA stability by Rho GTPase.* J Biol Chem, 1998. **273**(37): p. 24266-71.
8. Amano, M., et al., *Phosphorylation and activation of myosin by Rho-associated kinase (Rho-kinase).* J Biol Chem, 1996. **271**(34): p. 20246-9.
9. Kimura, K., et al., *Regulation of myosin phosphatase by Rho and Rho-associated kinase (Rho-kinase).* Science, 1996. **273**(5272): p. 245-8.
10. Wozniak, M.A., et al., *ROCK-generated contractility regulates breast epithelial cell differentiation in response to the physical properties of a three-dimensional collagen matrix.* J Cell Biol, 2003. **163**(3): p. 583-95.
11. Gauthier, N.C., T.A. Masters, and M.P. Sheetz, *Mechanical feedback between membrane tension and dynamics.* Trends Cell Biol, 2012. **22**(10): p. 527-35.
12. Tan, P.Y. and R. Zaidel-Bar, *Transient Membrane Localization of SPV-1 Drives Cyclical Actomyosin Contractions in the C. elegans Spermatheca.* Curr Biol, 2015. **25**(2): p. 141-51.
13. Ignarro, L.J., et al., *Endothelium-derived relaxing factor produced and released from artery and vein is nitric oxide.* Proc Natl Acad Sci U S A, 1987. **84**(24): p. 9265-9.
14. Archer, S.L., et al., *Nitric oxide and cGMP cause vasorelaxation by activation of a charybdotoxin-sensitive K channel by cGMP-dependent protein kinase.* Proc Natl Acad Sci U S A, 1994. **91**(16): p. 7583-7.
15. Carvajal, J.A., et al., *Molecular mechanism of cGMP-mediated smooth muscle relaxation.* J Cell Physiol, 2000. **184**(3): p. 409-20.
16. Bolotina, V.M., et al., *Nitric oxide directly activates calcium-dependent potassium channels in vascular smooth muscle.* Nature, 1994. **368**(6474): p. 850-3.
17. Lee, M.R., L. Li, and T. Kitazawa, *Cyclic GMP causes Ca2+ desensitization in vascular smooth muscle by activating the myosin light chain phosphatase.* J Biol Chem, 1997. **272**(8): p. 5063-8.
18. Zhang, Q., S. Bhattacharya, and M.E. Andersen, *Ultrasensitive response motifs: basic amplifiers in molecular signalling networks.* Open Biol, 2013. **3**(4): p. 130031.
